# Predicting the Learning Avoidance Motivation, Learning Commitment, and Silent Classroom Behavior of Chinese Vocational College Students Caused by Short Video Addiction

**DOI:** 10.3390/healthcare11070985

**Published:** 2023-03-30

**Authors:** Jian-Hong Ye, Zhen He, Xiantong Yang, Yi-Sang Lee, Weiguaju Nong, Jhen-Ni Ye, Chiung-Ling Wang

**Affiliations:** 1 Faculty of Education, Beijing Normal University, Beijing 100875, China; 2 National Institute of Vocational Education, Beijing Normal University, Beijing 100875, China; 3 Faculty of Psychology, Beijing Normal University, Beijing 100875, China; 4 Department of Industrial Education, National Taiwan Normal University, Taipei City 106010, Taiwan; 5 School of Education, Guangxi University of Foreign Languages, Nanning 530222, China; 6 Graduate Institute of Technological & Vocational Education, National Taipei University of Technology, Taipei City 106344, Taiwan

**Keywords:** ecological systems theory, person-process-content model, relying on short video apps, short film videos, short video problematic use

## Abstract

As short video addiction has gradually become an emerging Internet behavioral addiction, its negative impacts on the student population have been noticed and cannot be ignored. Based on a literature review, this study referred to the person-process-content framework and drew on the ecosystem theory to define the relationship between short video addiction, learning avoidance motivation, learning commitment, and silent classroom behavior by using structural equation modeling. This study recruited 1000 participants from Chinese vocational colleges to fill out questionnaires. With an effective recovery rate of 94.6%, there were 946 effective study participants comprising 445 males (47%) and 501 females (53%). After it was confirmed that the data passed the reliability and validity tests, structural equation model analysis was carried out. The study results showed that short video addiction was positively correlated with learning avoidance motivation, but negatively correlated with learning commitment; on the other hand, study avoidance motivation was negatively correlated with learning commitment, but positively correlated with silent classroom behavior. Meanwhile, there was a negative correlation between learning commitment and silent classroom behavior. As a result, the negative correlation between short video addiction and learning behavior needs attention from parents and teachers.

## 1. Introduction

### 1.1. The Influence of Short Videos on Learning

As a hedonic technology, short film videos (SFVs) such as TikTok, Kuaishou, and iXigua have attracted many users [1]. In the past few years, the number of short video users has increased dramatically, while also accelerating short video addiction as a new form of Internet addiction among the younger generations that cannot be ignored. To be more specific, SFVs have had a great negative impact on the student population. Therefore, exploring the issues regarding short video addiction has theoretical and practical significance.

Short video addiction is also known as pathological short video use or short video overuse. This is the situation when short video users cannot control their behavior properly and watch short videos excessively. This problematic usage is generally considered to be detrimental to users. Students in particular love short videos, but because of the immersive nature of the videos, many students can experience associated problems, and some even become addicted [2,3,4]. Therefore, Hong et al. pointed out that video addiction behaviors have had a very large negative impact on the study and life of students of different school ages [5]. “Tech Addictions” have become proprietary and are a popular topic of research in the information systems field; the main focus of the research to date has been on addiction and its antecedents [6]. However, Hong et al. suggested that follow-up research can adopt empirical research methods to explore the impact of video addiction on students’ learning [5]. Therefore, short video addiction is used as an independent variable of the research model to explore the relationship with the learning behavior of the student users in this study.

### 1.2. The Influence of Short Video Addiction from the Point of View of Ecological Systems Theory

To effectively determine the correlations between short video addiction and learning, a mature theory is required to support the findings. As one of the most well-known theoretical frameworks in social science, Brofenbrenner’s ecological systems theory (EST) is considered the most capable of describing and explaining the thoughts [7] and behaviors of individuals and groups within the specific context in which they live [8]. It can also provide an insightful perspective for determining the root cause of the problem [9]. Furthermore, the EST provides guidelines to higher education researchers and practitioners for how they can examine student development within their educational ecosystems [10]. The EST posits that each system has different norms, rules, and procedures that have direct relationships defined as the face-to-face events that students collect as experience, and these also affect how students understand the surrounding world [11]. The EST helps to explain students’ experience of using short videos and their own rules of learning, and so was adopted as the theoretical basis of this study.

Hong et al. further adopted the person-process-content (PPC) model in the EST to explore the relationship between addiction and learning according to the research purpose [5]. In this study, “person” is regarded as the short video addiction state of students, “process” is regarded as the pursuit and enthusiasm of students for learning tasks, and “content” is the behavioral performance in the classroom. Therefore, the EST’s PPC model was used to support the hypothetical model of this study.

### 1.3. Learning Avoidance Motivation

Furthermore, cognitive, affective, and behavioral aspects cannot be ignored when discussing student learning. Motivation itself can be treated as a representative factor of cognition, and its role in learning varies across studies [12]. For the students’ sakes, it is necessary to have positive learning motivation and avoid negative motivation, because motivation is significant for the completion of goal-directed behaviors [13]. Motivating behaviors can be driven to desired stimuli (approach motivation) or away from undesired stimuli (avoidance motivation) [14]. Hence, avoidance motivation is the motivation or direction of behavior away from negative stimuli (objects, events, possibilities) [15], and goal orientation that avoids performance is considered undesirable in an academic setting [14]. Avoidance learning motivation can be conceptualized as a negative learning behavior of avoiding studies. Considering the influences of avoidance learning motivation to learning enthusiasm and behavior, it was adopted as one of the variables of this study model.

### 1.4. Learning Commitment

Christenson et al. stated that commitment to learning contributes to individuals’ active participation in learning activities, which is considered to be the most important theoretical variable for understanding dropouts and facilitating school completion [16]. Rodríguez-Izquierdo also proposed that encouraging students’ learning commitment (academic commitment) is a student-centered teaching method implemented by universities, but students’ learning commitment does not always exist [17]. Therefore, it is important to identify the level of students’ commitment to their study. Learning commitment is conceptualized as a student’s long-term adherence to learning [18], which is an emotional manifestation. Meanwhile, students’ commitment is also considered as an attitude variable that affects their behavior [19]. Therefore, this study chose learning commitment as one of the variables in the study model.

### 1.5. Silent Classroom Behavior

Cultural characteristics in Asian societies are often cited as the main reason for this so-called silence and passivity [20]. Because Chinese students are influenced by traditional examination-oriented education, the teaching method is usually teacher-centered, and students listen to the teacher in class. The interaction between teachers and students is limited, and students often remain silent. However, classroom reticence behavior is an adverse factor affecting student-centered learning, which can be conceptualized as a kind of behavior in which students do not often interact with teachers or classmates in class and are quiet and reticent. This style of classroom performance often fails to bring about good learning outcomes [21], because students seem to be withdrawn and rarely speak, usually sit and observe, and show varying degrees of reserved or withdrawn attitudes. In group activities, students behave in an even more restrained and reserved way [22]. One of the greatest challenges, which results in low classroom participation and teacher frustration, is silence or failure to participate in classroom activities [23]. Therefore, silent classroom behavior was included as the outcome (dependent) variable of the study model.

### 1.6. Research Purpose and Contribution

Although there have been many studies focusing on college students’ persistence and educational outcomes, and most of the literature has studied various factors related to college students’ experience and environment, only a few studies have considered the impact of students’ psychological state and environmental background on their persistence decisions [24]. In order to expand the understanding of this topic, the purpose of this study was to verify the relationship between short video addiction, learning avoidance motivation, commitment, and silent classroom behavior of Chinese vocational college students through a structural equation model under the PPC framework of the ecosystem theory.

The problems caused by short video addiction have gradually attracted the attention of researchers from all around the world. Short video addiction is an emerging research field, but the current international empirical results are quite limited. This study helps to expand the international understanding of short video addiction research issues, which is the theoretical contribution of this study.

Avoidance motivation has generally been conceptualized in the past as the motivation for people to avoid exposing themselves to failure. However, in this study, the motivation to avoid learning was redefined. Students simply avoid academic activities or tasks, and do not take their own abilities into consideration. Such a concept will be more helpful for research to explore negative learning situations, including learning-weariness behaviors, which is one of the theoretical contributions of this study.

## 2. Theoretical Basis and Research Hypotheses

### 2.1. Research Model

Bronfenbrenner’s EST emphasizes the interrelationships between different processes and changes in their content [25]. The result of the interaction between the individual and the environment leads to one’s development [26]. Ozaki et al. believed that both internal and external environments have important influences on college students [24], so when researchers choose research concepts and theoretical frameworks, they will not limit students’ experience to the university campus. The results of interaction between people and the environment are called proximal processes. Using the proximal process concept, when college students find themselves immersed in the proximal process using short videos, they may not intend to actively learn, which may lead to their learning avoidance motivation, commitment to learning, and silent classroom behavior. Furthermore, placing students at the center of the EST framework helps to emphasize the importance of recognizing their individual needs, particularly their role as “active” participants in the learning process and how they affect the environment [27].

**Figure 1:**
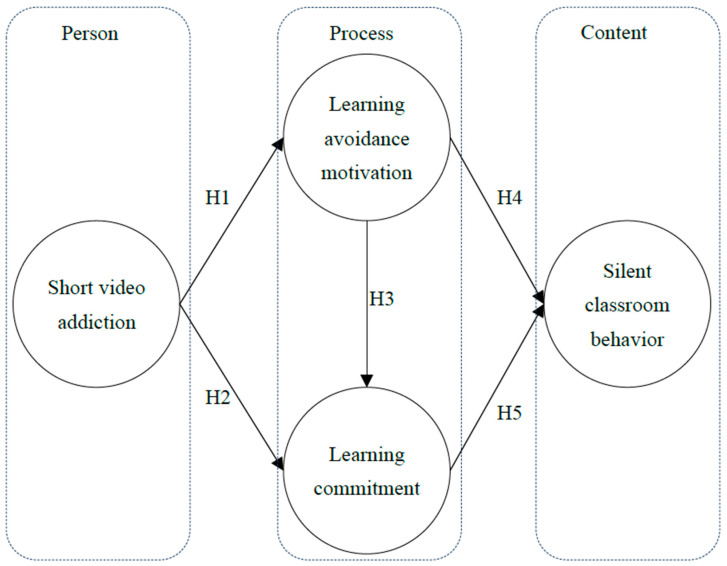
Research Model.

Based on the above literature review, the EST explains behavior and outcomes in different educational contexts. Therefore, this study adopted the framework of ecosystem theory to discuss the phenomenon of behavioral addiction and school connection. Under the framework of the PPC model of EST, this study constructed a research model and proposed five research hypotheses to explore the relationships between the short video addictions (person), avoidance learning motivation, commitment (process), and classroom silent acts (content). In other words, the relationship between short video addiction and learning avoidance motivation and learning commitment (H1 and H2), the relationship between learning avoidance motivation and learning commitment and silent classroom behavior (H3 and H4), and the relationship between Learning commitment and Silent classroom behavior (H5) were verified and viewed in this model. The research model is represented in Figure 1.

### 2.2. Research Hypothesis

#### 2.2.1. The Relationship between Short Video Addiction and Learning Avoidance Motivation

Currently, there are still very few empirical studies on short video addiction, but the negative impact of addiction on learning can be found from other studies on behavioral addiction. For example, studies have noted that problematic internet usage is negatively associated with learning motivation [28]. This phenomenon indicates that it is more difficult for students to organize their learning effectively while addicted to short videos [29]. Therefore, the excessive usage of the Internet is a factor that reduces the learning motivation of adolescents [30]. Furthermore, previous research has also found that school performance and mobile phone addiction have a strong negative correlation [31]. Studies have also found that despite the negative consequences for users, people are still willing to spend a great deal of time using short video apps [32]. In summary, when student users become addicted to short videos, they may not be interested in their studies and may have a reduced desire to learn. Therefore, this study adopted short video addiction to discuss the relationships between participants’ avoidance learning motivation. The hypothesis is as follows:

**H1:**  *Short video addiction is positively correlated with learning avoidance motivation.*

#### 2.2.2. The Relationship between Short Video Addiction and Learning Commitment

Users claim that gaming addiction can affect their learning and thinking [33]. At the same time, there is growing evidence that Internet usage is associated with negative academic outcomes [34]. In this situation, if technology is used inappropriately, it can have poor outcomes, including altering one’s daily life, even reducing sleep time, and inadvertent use can negatively affect students’ learning [35]. Users in an addictive state may experience more difficulties in maintaining attention, such as impaired concentration and inability to deal with distractions [2]. However, a growing number of teenagers are addicted to the virtual world created through mobile phones, and are even becoming addicted, so it has a serious negative impact on teenagers’ mentality, learning, and life [36]. Short video addiction can be described as an individual’s inability to control their short video app usage, despite the fact that such usage has negative psychological and social effects [37]. Therefore, the uncontrollable usage of short videos also has adverse consequences for students’ learning psychology. When student users become addicted to short videos, their engagement in learning may be reduced and they may lack enthusiasm for learning. Therefore, this study adopted short video addiction to explore the relationship between participants’ short video addiction and their learning commitment, hypothesized as follows:

**H2:**  *Short video addiction is negatively correlated with learning commitment.*

#### 2.2.3. The Relationship between Learning Avoidance Motivation and Learning Commitment

Motivation should be considered as a key factor in the learning process [38], because motivation affects students’ behavior and attitudes [30]. Motivational problems arise when individuals are unable to connect to the desired task or activity, which is defined as negative motivation or no-motivation [39]. Avoidance motivation is limited as a dependent variable because it can only lead to the absence or presence of negative outcomes by its very nature [15]. For students with high rates of task avoidance, “success” was defined in terms of minimal work expenditures rather than any external or personal measure of ability [14]. Therefore, some studies have pointed out that a high level of avoidance orientation is not conducive to students’ personal learning [40]. Based on the above, when student users deliberately avoid learning tasks, their enthusiasm for learning may also decrease. Therefore, this study adopted avoidance learning motivation to explore the relationship between participants’ learning commitment; the hypothesis is as follows:

**H3:**  *Learning avoidance motivation is negatively correlated with learning commitment.*

#### 2.2.4. The Relationship between Avoidance Learning Motivation and Silent Classroom Behavior

Motivation is closely related to student engagement in the classroom, including some school atmosphere such as educational aspirations, enjoyment of school, academic learning, and academic achievement [41]. In the field of motivational research, the study of stimuli and responses is carried out to assess the types and causes of stimuli that elicit appropriate responses to various learning situations [38]. For example, students with strong task avoidant tendencies may also not proactively seek advice from teachers and respond to assignments in an uninspired manner [40]. Especially in high-risk situations, students usually have negative learning motivation, which is detrimental to the preparation for learning [42]. If students actively participate in the learning process, a more positive impact is generated [43]; on the contrary, if students show negative motivation, they will certainly not want to invest in learning. When student users intentionally avoid learning tasks, their performance in class may be less lively or colder. Therefore, this study adopted avoidance learning motivation to explore the relationship between participants’ silent behavior in the classroom; the hypothesis is as follows:

**H4:**  *Avoidance learning motivation is positively correlated with silent classroom behavior.*

#### 2.2.5. The Relationship between Learning Commitment and Silent Classroom Behavior

Learner commitment may be a more critical factor in achieving learning goals than learner competence [44], because the level of commitment affects students’ learning methods, which in turn affects their academic achievement [45]. Therefore, commitment is considered to be a key factor affecting student performance [46]. Because students adjust their learning commitments based on real-time assessments of events, these changes in learning commitments have corresponding effects on student behavior and achievement outcomes [47]. While some students try to do their best in situations of achievement, others will appear disengaged and just want to end the situation and get it done in an easy way [14]. Therefore, having a high level of commitment to learning enhances the elements of learning [43] and is of great importance to student retention, achievement, and success [47]. Based on the above, when student users are highly enthusiastic about learning, they may also participate more actively in class. This study adopted learning commitment to explore the relationship between participants’ silent classroom behavior; the hypothesis is as follows:

**H5:**  *Learning commitment is negatively correlated with silent classroom behavior.*

## 3. Materials and Methods

### 3.1. Study Design

This study adopted the snowball sampling method and distributed online questionnaires through the Questionnaire Star platform. The questionnaire was initially forwarded to vocational college teachers in six provinces of China (Guangxi, Guangdong, Hainan, Jiangsu, Zhejiang, Hebei) and handed out to the participants in their classes to fill out the questionnaires. It was then once again forwarded to other classmates at the same schools. Between 15 and 26 December 2022, the number of returned questionnaires was 1000.

Because the participants in this study were all adult students and the data were collected anonymously through the Internet, there was no mandatory requirement for a research ethics review. However, this study still followed international research ethics norms. During the process of recruiting participants for this study, recruitment publicity was provided, which explained the purpose of the study, data usage, participant privacy, data protection, etc. The relevant information was also revealed in the informed consent statement on the first page of the online questionnaire. Only if the participants agreed to participate in the research could they fill in the online questionnaire.

### 3.2. Participants

The number of participants in this study (returned questionnaires) was 1000, and a total of 54 invalid data were deleted (incomplete questionnaires). The effective research participants therefore numbered 946, giving an effective recovery rate of 94.6%. There were 445 males (47%) and 501 females (53%). There were 179 first-year students (18.9%), 434 second-year students (45.9%), 274 third-year students (29%), and 59 fourth-year students (6.2%); 78 respondents (8.3%) watched short videos 1–3 days a week, 163 (17.2%) watched 4–6 days a week, and 705 (74.5%) watched every day. On average, 66 (7%) watched short videos for less than 1 h a day, 505 (53.4%) watched for 1–3 h a day, 261 (27.6%) watched for 3–5 h a day, and 114 watched for more than 5 h a day (12%), as shown in Table 1. The mean age of participants was 19.47 years (SD = 1.02).

### 3.3. Measurement

The measurements of this study included short video addiction, learning avoidance motivation, learning commitment, and silent classroom behavior. The item sources of each variable include references, modifications of questionnaires designed by other scholars, and a scale compiled for this study. When the measurement design of this study was completed, three experts in educational psychology conducted a content validity review of the questionnaire to confirm whether the content of the measurement could effectively reflect the purpose of this study. The questionnaire evaluation standard adopted a 5-point Likert scale, where 1 to 5 represented strongly disagree to strongly agree, respectively. The higher the score, the higher the degree of approval of the participant. The scales are described in the following sections. The complete questionnaire can be found in the Appendix A. 

#### 3.3.1. Short Video Addiction Scale

This study adopted the Short Video Addiction Scale of Ye et al. [48], with a total of 10 items. Example questions include: “I will put down the things that should be completed or executed and spend time watching short videos” and “I will still remember the content of short videos after turning off the platform.” The higher total score related to the state of being unable to watch short videos autonomously represented a higher degree of addiction as perceived by the individual.

#### 3.3.2. Avoidance Learning Motivation Scale

This study referred to the Question Avoidance Motivation Scale of Hong et al. [49], and revised the Avoidance Learning Motivation Questionnaire with a total of eight questions. Example questions are: “I don’t like to spend energy on learning tasks” and “When I am doing homework, I will try to choose the easiest way to do it.” Because this scale measured students’ motivation to avoid learning tasks when they were studying, a higher total score represented a higher degree of learning avoidance motivation perceived by the individual.

#### 3.3.3. Learning Commitment Scale

This study referred to and edited the Learning Commitment Scale of Rodríguez-Izquierdo (2020), with a total of nine items. Example questions are: “I find my studies imbued with meaning and purpose” and “I am proud to be in college.” Because the scale measured students’ persistence and identification with their own learning activities, a higher total score represented a higher degree of personal perceived commitment.

#### 3.3.4. Silent Classroom Behavior Scale

In this study, a scale was developed based on silent classroom behavior, with a total of seven items. Example questions include: “I don’t want to participate in classroom activities during class” and “When I encounter problems in class, I will choose to keep silent and don’t want to ask the teacher.” because this scale measured students’ self-esteem and silent behavior performance in class, a higher total score represented a higher level of silent behavior as perceived by the individual.

### 3.4. Statistical Analysis 

Structural Equation Modeling (SEM) is a general statistical modeling tool used to describe a large number of statistical models for assessing the validity of substantial theories with empirical data [50], which is also applicable to model and theory testing and scale development [51]. Specifically, this technique combines regression, correlation, and factor analysis simultaneously to address important problems in the social sciences, biological sciences, and humanities. Its greatest strength is the ability to manage measurement error, which is one of the greatest limitations of most studies, and thus SEM is now widely used in research [52]. As a result, the structural equation modeling software AMOS 20.0 was used to verify the paths of the research hypotheses. 

## 4. Results

### 4.1. Measurement Model Evaluation

Measurement model analysis helps us understand how well the items fit each variable. Therefore, the measurement model evaluation for this study was a confirmatory factor analysis (CFA) using AMOS. The degree of fit of each measurement model is shown in Table 2. During this evaluation phase, items with a Factor Loading (FL) lower than 0.50 were deleted, as such a low FL represents poor validity. The result of the deletion of questions was that short video addiction was reduced from ten to six questions, avoidance learning motivation was reduced from eight to five questions, learning commitment was reduced from nine to six questions, and silent classroom behavior was reduced from seven to four questions.

### 4.2. Reliability and Validity Analysis

In quantitative research, reliable and valid data are required to ensure that the analysis results can be trusted. Table 3 shows the mean (M), standard deviation (SD), Cronbach’s α and FL for each variable. In accepted academic standards, the values of Cronbach’s α should be higher than 0.70 to ensure good reliability. The values of FL should be higher than 0.50 to ensure good validity. In this study, the Cronbach’s α value of the variables ranged from 0.89 to 0.94, and thus met the recommended standards. The FL values ranged from 0.76 to 0.90, as shown in Table 3.

### 4.3. Related Analysis

To confirm the correlation between variables, the Pearson Correlation analysis technique was applied. From Table 4, it can be seen that the results showed significant correlations among short video addiction, avoidance learning motivation, learning commitment, and silent classroom behavior. 

### 4.4. Overall Fitness Evaluation

The degree of fit of the hypothesis model determines the validity of the model. Evaluation of parameter estimates was used in this study to assess model fit. The fitting index values of this study were χ^2^ = 699.59, *df* = 184, χ^2^/*df* = 3.80, RMSEA = 0.05, GFI = 0.93, AGFI = 0.92, NFI = 0.96, NNFI = 0.96, CFI = 0.97, IFI = 0.97, RFI = 0.95, PNFI = 0.84, and PGFI = 0.74. The results showed that the data fit well with the model.

### 4.5. Research Model Validation

**Figure 2:**
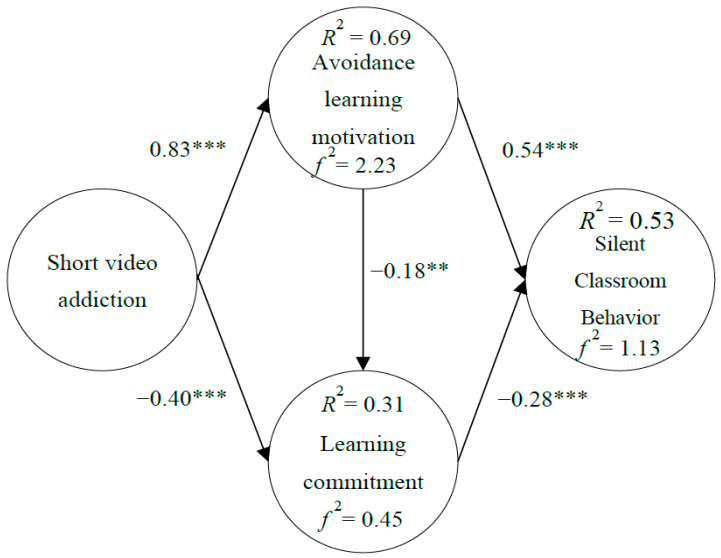
Research Model Validation. ** *p* < 0.01, *** *p* < 0.001.

Whether the path assumed by the research can be established needs to be verified by the model. This study adopted SEM to test the hypothesized model. The results showed that short video addiction was positively correlated with learning avoidance motivation (β = 0.83 ***), short video addiction was negatively correlated with learning commitment (β = −0.40 ***), avoidance learning motivation was negatively correlated with learning commitment (β = −0.18 **), learning avoidance motivation was positively correlated with silent classroom behavior (β = 0.54 ***), and learning commitment was negatively correlated with silent classroom behavior (β = −0.28 ***). It can be seen from Figure 2 that the five research hypotheses proposed in this study were all supported. That also meant the higher the participants’ level of short video addiction tendency, the higher their avoidance learning motivation was, and the lower their level of learning commitment was. The higher the students’ degree of avoidance learning motivation, the lower their learning commitment, and the more frequently silent classroom behavior appeared. However, the higher the students’ learning commitment level, the less silent classroom behavior happened. In addition, the explanatory power of short video addiction to learning avoidance motivation was 69%, and ƒ^2^ was 2.23; the explanatory power of short video addiction and learning avoidance motivation to learning commitment was 31%, and ƒ^2^ was 0.45; the explanatory power of avoidance learning motivation and learning commitment to silent classroom behavior was 53%, and ƒ^2^ was 1.13. In other words, each model path had good explanatory power and effect size, as shown in Figure 2.

### 4.6. Analysis of Indirect Effects

It could be seen from Table 5 that only short video addiction and silent classroom behavior had positive indirect effects, and the analysis of the other two indirect effects had not reached a significant level and had no statistical significance.

## 5. Discussion

### 5.1. Short Video Addiction Is Positively Correlated with Learning Avoidance Motivation

The results of the study showed that short video addiction was positively correlated with avoidance learning motivation, so H1 was supported. It could be said that when student users have short video addiction, they are not interested in their studies and reduce their desire to participate in learning. The reason for this phenomenon is that students who are addicted to short videos hope to keep watching short videos to satisfy their own entertainment, so they do not want to learn, or avoid learning tasks. The results of this study are also similar to previous studies, such as those of Reed and Reay, Truzoli et al., and Demir and Kutlu, all of which confirmed that Internet addiction has a negative impact on academic motivation [28,29,30]. This also makes it difficult for students with Internet addiction to carry out their learning activities effectively. In addition, the research of Bai et al. found that there was a strong negative correlation between mobile phone addiction and students’ school performance [31].

### 5.2. Short Video Addiction Is Negatively Correlated with Learning Commitment

The results of the study showed that short video addiction was negatively correlated with learning commitment, so H2 was supported. In other words, when student users become addicted to short videos, their recognition and persistence in learning decrease, and they lack enthusiasm for learning. The reason for this is that students who are addicted to short videos are already attached to the content of short videos and spend most of their attention on short videos. As a result, it is easy to lack enthusiasm for learning. While the results of this study coincided with past research results, such as Mo et al., there is also increasing evidence that Internet use is associated with negative academic outcomes [34]. The surveys by Zhai et al. indicated that users claimed that gaming addiction affected their learning and thinking [33]. The study by Chen et al. found that users in an addictive state had impaired concentration and an inability to process distractions [2]. Nevertheless, there are still many adolescents who are addicted to the virtual world, which has a serious negative impact on the mentality, study, and life of adolescents [36].

### 5.3. Learning Avoidance Motivation Is Negatively Correlated with Learning Commitment

The study results showed that learning avoidance motivation was negatively correlated with learning commitment, so H3 was supported. In other words, when student users deliberately avoid learning tasks, their sense of identity and persistence in learning also decrease. The reason for this is that when students have the idea of avoiding learning tasks, they will not want to learn more, and their enthusiasm for learning will also decrease. The findings of this study also have many similarities with previous studies. As proposed by Demir and Kutlu, motivation is a key factor influencing student behavior and attitudes [30]. Motivational problems arise when individuals fail to connect with the task or activity to be performed [39]. Elliot stated in his research that avoidance motivation due to its nature is limited as the dependent variable, because it can only happen in the absence or existence of negative outcomes [15]. Hirschfeld et al. also clearly stated that when students have a high level of avoidance orientation, it is detrimental to their personal learning [40].

### 5.4. Avoidance Learning Motivation Is Positively Correlated with Silent Classroom Behavior

The study results showed that avoidance learning motivation was positively correlated with silent classroom behavior, so H4 was supported. When student users deliberately avoid learning tasks, their performance in the classroom is less lively or more indifferent. When students already have the idea of avoiding learning tasks, they naturally do not want to actively participate in the interaction in the classroom. This is consistent with Collie and Martin’s study (2019), which found that motivation is closely related to student engagement in the classroom, educational aspirations, enjoyment of school, academic learning, and academic achievement [41]. Additionally, Ferreira et al. indicated that the type and cause of motivation can elicit appropriate stimulus responses to various learning situations [38]. Some scholars have found that positive motivation brings positive results, and negative motivation brings negative results. Che Ahmad et al. stated that if students actively participate in the learning process, it has a more positive impact on their learning [43]. On the other hand, completely different results will be obtained in the opposite situation. Research by Hirschfeld et al. found that students with strong avoidance tendencies may not actively seek advice from teachers, and may respond to assignments in an uninspired manner [40]. Harlen and Deakin Crick also believed that when students have passive learning motivation, it hinders their learning [42].

### 5.5. Learning Commitment Is Negatively Correlated with Silent Classroom Behavior

The research results showed that learning commitment was negatively correlated with silent classroom behavior, so H5 was supported. In other words, when student users are highly enthusiastic about learning, they are more active in participating and publishing in class. When students are passionate about learning, they also want to have more exchanges with teachers or classmates in class to gain more knowledge and experience. This is consistent with the views of some scholars. As indicated by Che Ahmad et al., Kim et al., and Mart, learner commitment may be a more critical factor in achieving learning goals than learner ability [43,44,45,46]. Wilkins et al. further explained that this is because the level of commitment will affect students’ learning methods, which in turn will affect their academic achievement [45]. Students with high levels of commitment try to do their best in situations of achievement, but students with lower levels of commitment show disengagement and just want to end the situation quickly [14]. Griep argued that these changes in commitment to learning have corresponding effects on student behavior and achievement outcomes [47].

### 5.6. Research Limitations and Recommendations for Future Research

This study has several limitations. First, this was a cross-sectional design study using questionnaires to confirm the correlation between short video addiction and learning behavior. Therefore, the causal relationship between variables cannot be confirmed in this study. Follow-up research can use a longitudinal design to confirm the causality between short video addiction and different learning experience disorders. This can also help understand the impact of another element of EST, “time,” on students’ learning and development. Personal in-depth interviews can also be used to learn more about the thoughts and learning experiences of student users.

Second, although the issue of short video addiction has been a concern for several years, related research is only in its infancy. Therefore, follow-up researchers can explore the influence of short video addiction on student groups from different theoretical perspectives or different analysis methods. In addition, the participants in this study were Chinese vocational school students, which may limit the generalizability of the findings. Follow-up research can recruit students at different stages of study to participate in the research to expand the interpretable scope of the research.

Third, the duration of short video use does not necessarily mean that the user has become addicted, and it is also meaningful to confirm the purpose of the use. Therefore, in follow-up research, the motivation for using short videos can be used to explore the causes of short video addiction. More details are needed to confirm whether the participants have short video addiction, and which motivations are more likely to contribute to short video addiction.

Fourth, bad or negative learning behavior or mentality is an educational issue that needs to be taken seriously. However, behind the problem of poor online or offline learning effects, there are still many factors or consequences worth exploring in detail, or corresponding solutions; for example, what other factors trigger students’ silent classroom behavior, and how to improve such behavior. These are worthy of further exploration in follow-up research.

## 6. Conclusions and Recommendations

### 6.1. Conclusions

Under the PPC framework based on ecosystem theory, the structural equation modeling technique was used to explore the relationship between short video addiction, learning avoidance motivation, commitment, and classroom silent behavior. The research results showed that: (1) Short video addiction was positively correlated with learning avoidance motivation, but negatively correlated with learning commitment. (2) Study avoidance motivation was negatively correlated with learning commitment, but positively correlated with classroom silent behavior. (3) There was a negative correlation between learning commitment and classroom silent behavior. The results showed that there was a negative correlation between short video addiction and students’ academic behavior.

From the perspective of contemporary education, silent classroom behavior is regarded as very poor learning performance. However, previous studies on silent classroom behavior mainly focused on how to improve students’ classroom silence in the context of language classes. This directly leads to neglecting the silent behavior of students in the classroom that should be paid attention to in any classroom. The discovery of the relationship between students’ attitudes and motivations and silent classroom behavior help to expand our understanding of silent classroom behavior.

### 6.2. Recommendations

Short video addiction, like other types of Internet addiction, is a problem that needs to be taken seriously by parents and educators because the research results show that short video addiction will have a great impact on the learning situation, including causing more negative psychological perceptions and behavior. However, it seems to be very difficult to monitor the short video app usage behavior of adult students. Therefore, it is suggested that parents and educators should appeal to student users to cultivate interest in various leisure activities and avoid relying too much on short videos for entertainment experience, so as to avoid relying on short video apps.

For students with high levels of avoidant learning motivation, teachers should help them find achievement goals to drive their desire to learn and let them gradually develop enthusiasm for learning activities. At the same time, teachers should also interview students with high levels of avoidance learning motivation to find out the reasons for their avoidance of learning tasks, in order to better help them be willing to learn.

It is generally accepted that taciturn students usually speak less and exhibit more negative forms of behaviors [53]. Therefore, teachers should adopt more learning-centered teaching methods in the classroom so that students can become the protagonists of learning. More attention can be given to students who are not highly involved in courses, and group activities can also be added to increase student participation.

## Figures and Tables

**Table 1:** Participant Profile Analysis.

Variables	Classifications
Gender	445 males (47%) 501 females (53%).
Grades	179 first-year students (18.9%), 434 second-year students (45.9%), 274 third-year students (29%) 59 fourth-year students (6.2%);
Average number of days per week	78 respondents (8.3%) watched short videos 1–3 days a week 163 (17.2%) watched 4–6 days a week 705 (74.5%) watched every day.
Average number of hours per day	66 (7%) watched short videos for less than 1 h a day 505 (53.4%) watched for 1–3 h a day 261 (27.6%) watched for 3–5 h a day 114 watched for more than 5 h a day (12%).

**Table 2:** First order confirmatory analysis.

Adaptability	Critical Value	Short Video Addiction	Avoidance Learning Motivation	Learning Commitment	Silent Classroom Behavior
χ^2^	---	39.4	5.8	32.2	6.3
*df*	---	9	5	9	2
χ^2^/*df*	<5	4.38	1.16	3.66	3.15
RMSEA	<0.10	0.06	0.01	0.05	0.05
GFI	>0.80	0.99	0.99	0.99	0.99
AGFI	>0.80	0.97	0.99	0.97	0.98
FL	>0.50	0.75~0.88	0.69~0.92	0.60~86	0.83~0.96
*t*	>3	29.91~40.56	23.37~43.82	17.50~39.18	35.68~45.54

**Table 3:** Reliability and validity analysis.

Construct	M	SD	α	FL
	---	---	>0.70	>0.50
Short Video Addiction	2.43	0.86	0.93	0.83
Avoidance Learning Motivation	2.49	0.85	0.90	0.81
Learning Commitment	3.36	0.70	0.89	0.76
Silent Classroom Behavior	2.58	0.811	0.94	0.90

**Table 4:** Pearson Correlation analysis.

Construct	1	2	3	4
1. Short Video Addiction	1			
2. Avoidance Learning Motivation	0.77 ***	1		
3. Learning Commitment	−0.50 ***	−0.45 ***	1	
4. Silent Classroom Behavior	0.64 ***	0.62 ***	−0.51 ***	1

*p* *** < 0.001.

**Table 5:** Analysis of indirect effects.

	Short Video Addiction		Avoidance Learning Motivation	
	β	95% CI	β	95% CI
Learning Commitment	−0.15	[−0.37, 0.01]		
Silent Classroom Behavior	0.604 ***	[0.54, 0.67]	0.05	[−0.01, 0.01]

*** *p* < 0.001.

## Data Availability

The original contributions presented in the study are included in the article; further inquiries can be directed to the corresponding author.

## Appendix A. Questionnaire

**No.**	**Items**
	Short Videos Addiction Scale
1.	I watch short videos for longer than I would have expected.
2.	I will put down the things that should be completed or executed and spend time watching short videos.
3.	I get more excitement or anticipation from watching short videos than I do from other human interactions.
4.	I get complained about or blamed for watching short videos.
5.	I would be late for school or leave early or be absent because of watching short videos.
6.	I get a drop in grades due to watching short videos.
7.	I get annoyed and angry if someone interrupts me while I’m watching short videos.
8.	I would sacrifice my night’s sleep for watching short videos.
9.	I will still remember the content of short videos after turning off the platform.
10.	I get depressed when not watching short videos.
	Avoidance Learning Motivation Scale
1.	I don’t like to spend energy and time on learning tasks.
2.	I often want to avoid learning tasks.
3.	I don’t want to put in more effort to get better academic performance.
4.	When I need to repeatedly correct my homework, I feel tempted to give up.
5.	While doing homework, I try to choose the easiest way to do it.
6.	When a classmate invites me to study together, I choose to reject the invitation.
7.	When I encounter difficulties in my studies, I am inclined to give up.
8.	When I encounter something I don’t understand while studying, I don’t take the initiative to find the answer.
	Learning Commitment Scale
1.	Doing homework makes me feel energetic.
2.	Studying or taking classes makes me feel physically strong and powerful.
3.	When I wake up in the morning, I want to go to class or study.
4.	I find my studies imbued with meaning and purposes.
5.	My studies inspire me.
6.	I am proud to be in college.
7.	While doing tasks related to my studies, I feel that time passes quickly.
8.	Doing homework is easy for me to get immersed in.
9.	When I study, I tend to forget everything happening around me.
	Silent Classroom Behavior Scale
1.	I don’t feel like speaking up and expressing opinions during class.
2.	I don’t want to participate in any activities in the class.
3.	I don’t want to interact with my classmates while I’m in class.
4.	I don’t want to interact with the teacher while I’m in class.
5.	When I encounter problems in class, I prefer to keep silent rather than ask my classmates for advice.
6.	When I encounter problems in class, I keep silent rather than ask the teacher.
7.	When my classmates don’t understand something, I will remain silent and not take the initiative to help them, even though I know the answer.
